# Percutaneous Atherectomy Versus Balloon Angioplasty/Stenting in the Treatment of Femoropopliteal Arterial Occlusive Disease

**DOI:** 10.3390/jcm14196926

**Published:** 2025-09-30

**Authors:** Hyangkyoung Kim, Taeseung Lee, Young Sun Yoo, Seung-Kee Min, Jin mo Kang, Jin Hyun Joh

**Affiliations:** 1 Department of Surgery, Ewha Womans University College of Medicine, Ewha Womans University Medical Center, Seoul 07985, Republic of Korea; 2 Department of Surgery, Seoul National University Bundang Hospital, Seongnam 13620, Republic of Korea; 3 Department of Surgery, Chosun University College of Medicine, Gwangju 61452, Republic of Korea; 4 Department of Surgery, Seoul National University College of Medicine, Seoul 03080, Republic of Korea; 5 Department of Surgery, Gachon University Gil Medical Center, Inchon 21565, Republic of Korea; 6 Department of Surgery, Kyung Hee University Hospital at Gangdong, Seoul 05278, Republic of Korea

**Keywords:** atherectomy, angioplasty, balloon, stents, peripheral arterial disease

## Abstract

**Objectives**: Atherectomy use for the treatment of femoropopliteal lesions has significantly increased. This study aimed to assess the clinical benefits of percutaneous atherectomy (PA) over balloon angioplasty and/or stenting (PTA ± stent) for femoropopliteal arterial disease using a nationwide prospective multicenter registry. **Methods**: Using data from the Damoeum registry of the Korean Society for Vascular Surgery, we identified patients with revascularization due to lower-extremity arterial disease. After excluding patients who underwent open and hybrid revascularization, we compared the clinical outcomes of the patients in the PA group versus the PTA ± stent group. We investigated the target lesion patency and functional and safety outcomes during the follow-up. **Results**: A total of 424 patients were included in the final analysis: 90 in the PA group and 334 in the PTA ± stent group. There were 344 men and 79 women (mean age: 71.1 years). The preprocedural ankle–brachial index (ABI) was significantly increased in both groups (*p* = 0.015). When we compared 90 patients of the PA group and 270 patients of the matched PTA ± stent cohort (1:3 propensity-matched cohort), the overall 1-year primary patency rate was not significantly different (83.8% vs. 80.0%; *p* = 0.895). However, the PA group showed a significantly lower risk of occlusion compared with the PTA ± stent group during the follow-up (adjusted HR: 0.01; *p* < 0.001). Overall mortality was similar in the two groups (*p* = 0.695). **Conclusions**: The use of atherectomy was not associated with improvement in target lesion patency. However, the use of atherectomy devices demonstrated a significant reduction in target lesion occlusion during the follow-up.

## 1. Introduction

The femoropopliteal segment is the most common site of atherosclerotic involvement in the lower extremity, accounting for more than 50% of cases. Endovascular approaches have obtained the popularity to revascularize this segment in patients with disabling claudication and chronic limb-threatening ischemia (CLTI) because of their decreased morbidity [1,2]. Although the decision regarding endovascular revascularization or bypass surgery depends on a comprehensive evaluation of anatomical and patient characteristics, the technological advances of endovascular revascularization have allowed an acceptable approach in increasingly complex lesions. However, despite its advancement, high rates of restenosis and reintervention remain significant challenges due to unique mechanical stresses in this segment, including flexion/extension, compression/elongation, and torsion, as well as high prevalence of chronic total occlusions, diffuse plaque, and heavy calcification [3].

Percutaneous transluminal angioplasty (PTA) with or without stenting remains the cornerstone of endovascular treatment for femoropopliteal disease. Stent placement improves acute luminal gain and decreases the risk of elastic recoil or flow-limiting dissection after angioplasty [4]. However, long-term durability remains limited by restenosis, in-stent restenosis, and the risk of stent fracture in the femoropopliteal artery, which is subject to significant mechanical stress during limb movement [5,6]. These limitations have driven ongoing interest in adjunctive techniques such as atherectomy to optimize vessel preparation and improve outcomes [7].

Percutaneous atherectomy (PA) permits the removal of calcified and fibrotic plaques rather than pressing them against the arterial wall with conventional balloon angioplasty or stent placement. PA provides maximal luminal gain without barotrauma and subsequently facilitates low-pressure balloon angioplasty, minimizing the likelihood of dissection and the need for stent placement. In addition, it increases simultaneous drug delivery into the vessel wall when a drug-coated balloon (DCB) is used [8]. As a result, PA has frequently been used for femoropopliteal lesions in the United States (US). A nationwide US analysis of the use of endovascular devices for femoropopliteal lesions between 2011 and 2019 showed that angioplasty alone and bare-metal stent implantation decreased during that period while the use of atherectomy increased from 33% in 2011 to 53% in 2019 [9].

Several studies in this segment demonstrated satisfactory mid-term results after PA compared to other endovascular techniques [10,11]. However, the outcomes associated with the use of PA have not been extensively studied in a real-world setting. The purpose of this study is to analyze the clinical outcomes after endovascular revascularization of the femoropopliteal segment using an ongoing nationwide multicenter registry.

## 2. Materials and Methods

### 2.1. Database

The Korean Society for Vascular Surgery (KSVS) launched the DAMOEUM registry in July 2020 as a nationwide prospective multicenter observational study of patients with lower-extremity artery disease who have undergone open, endovascular, and hybrid revascularization. The de-identified registry contains pre-, intra-, and postoperative variables from 21 centers throughout the country. Each institution obtained informed consent and received approval from its respective Institutional Review Board (KHNMC 2020-10-033-004), with a notable feature of the registry being the rigorous on-site data audit, entry of procedure records, and verification performed by the study staff.

### 2.2. Population

After approval of the study proposal from the Health Information Technology Committee of the KSVS, the full data of lower-extremity arterial revascularization between 2021 and 2023 were received. From these data, a list of patients with femoropopliteal revascularization was selected. Among these patients, we excluded the patients who underwent bypass surgery and hybrid revascularization. Finally, we analyzed two groups of patients who underwent percutaneous atherectomy (PA group) over balloon angioplasty and/or stenting (PTA ± stent group).

### 2.3. Variables

Baseline characteristics, including demographics (age, sex, body mass index), comorbidities (diabetes, hypertension, hyperlipidemia, coronary artery disease, chronic kidney disease, pulmonary disease, smoking status), preoperative medications (antiplatelet, anticoagulant), clinical severity with Rutherford category, and functional status (Walking Impairment Questionnaire and American Society of Anesthesiologists scores), were evaluated. The anatomic region of the target limb was divided into 3 segments, including inflow (aortoiliac), femoropopliteal, and outflow (below-the-knee) lesions. Anatomical grading of each lesion was used with the TransAtlantic InterSociety Consensus (TASC). Each lesion was classified as a de novo lesion, in-stent restenosis, or re-intervention. The treated lesion sites were classified as proximal, mid-, and distal for the superficial femoral artery (SFA). The popliteal arterial lesion was divided into the P1 (from the channel of the adductor muscles to the upper border of the patella), P2 (from the upper border of the patella to the joint line), and P3 (from the joint line to the emergence of the anterior tibial artery) segments. The calcification grade was classified with the involvement degree of circumference. Intraoperative variables, including procedure details and immediate procedure outcomes, were estimated. In the atherectomy group, the following devices were used: the Jetstream™ Atherectomy System (Boston Scientific, Marlborough, MA, USA) and the SilverHawk^®^, TurboHawk™, and HawkOne™ directional atherectomy systems (all Medtronic, Minneapolis, MN, USA). In the PTA ± stent group, the stents included Zilver^®^ PTX (Cook Medical, Bloomington, IN, USA), Supera™ (Abbott Vascular, Santa Clara, CA, USA), Eluvia™ (Boston Scientific, Marlborough, MA, USA), and EverFlex™ (Medtronic, Minneapolis, MN, USA). For drug-coated balloon (DCB) angioplasty, IN.PACT™ Admiral™ (Medtronic, Minneapolis, MN, USA), Ranger™ (Boston Scientific, Marlborough, MA, USA), and Lutonix^®^ (BD, Franklin Lakes, NJ, USA) were used. Postoperative surveillance information was evaluated using the functional and hemodynamic outcomes and the occurrence of any complications.

### 2.4. Outcomes

Procedural success was defined as the successful revascularization of the target lesion with ≤30% residual stenosis and without complications. Patency was defined as the presence of one or more of the following items during the follow-up period: unlimited blood flow without any signs of stenosis or occlusion or with less than 50% stenosis within the 5 mm borders adjacent to the target lesion, with imaging tests including duplex ultrasound, computed tomogram angiography (CT), magnetic resonance image angiography (MRA), and contrast angiography, and an increased ABI by more than 0.15.

### 2.5. Statistical Analysis

Normally distributed continuous variables were expressed as means ± standard deviation, while non-normally distributed continuous variables were expressed as medians with 25–75% quartiles. Categorical variables were expressed as numbers and percentages. The unpaired *t*-test, the Mann–Whitney U test, or Pearson’s Chi-square test were used for appropriate comparisons. Propensity score matching was employed to minimize the effect of baseline demographics. The matched cohort was selected from the control group based on propensity score similarities using a 1:3 ratio for the following covariates: age, sex, indication, and diabetes. Primary patency was determined using the Kaplan–Meier method and compared using log-rank tests. A Cox proportional hazard regression model was used to determine the adjusted hazard ratio (HR) with corresponding 95% confidence intervals (CIs). For comparisons with the matched cohort, clustered log-rank tests and Cox regression with marginal modeling were employed. A significance level of *p* < 0.05 was defined. Statistical analysis was performed using R software, version 4.2.2 (R Foundation for Statistical Computing, Vienna, Austria), and IBM SPSS Advanced Statistics, version 23.0 (IBM Corp., Armonk, NY, USA, released 2015).

## 3. Results

### 3.1. Patient Characteristics

Between 2021 and 2023, a total of 1057 patients underwent revascularization due to lower-extremity arterial disease. Among them, revascularization in the femoropopliteal segment was performed in 549 patients. We excluded 109 patients with bypass surgery and 16 patients with hybrid surgery. Finally, we analyzed 424 patients who underwent endovascular procedures (Table 1). The median age was 72 years and there were 345 (81.4%) male patients. We compared two groups that underwent PA (90 patients) and PTA ± stent (334 patients). Compared with the PTA ± stent group, patients in the PA group were younger (69 vs. 72 years; *p* = 0.016); had a higher rate of pulmonary diseases, including chronic obstructive lung disease (43.3% vs. 25.2%; *p* = 0.001); and had a higher rate of preprocedural medication of any kind of antiplatelet (71.1% vs. 61.4; *p* = 0.001) or anticoagulant (10.0% vs. 8.7%; *p* = 0.001). Indications for the revascularization in the PA and PTA ± stent groups were disabling claudication (64.4% vs. 31.7%) and critical limb-threatening ischemia (35.6% vs. 68.3%), respectively (*p* < 0.001).

### 3.2. Anatomical Characteristics

The detailed anatomical characteristics are demonstrated in Table 2. The most common lesion type was de novo lesion (78.5%), followed by in-stent restenosis (9.7%) and repeated intervention (9.2%). The types of the target lesions were not statistically significant in two groups (*p* = 0.725). Lesion sites were divided into six positions: proximal, mid, and distal portions for the SFA and P1, P2, and P3 for the popliteal artery. If the treated lesion sites were combined, each site was evaluated as treated. The most commonly treated site was mid-SFA (51.2%). However, the most common treated site in PA group was the proximal SFA (55.6%), and in the PTA ± stent group, it was mid-SFA (51.5%). The treated lesion site was similar in two groups (*p* = 0.745). The complex TASC C and D lesions were treated in 33.4% of the PA group and 29.7% of the PTA ± stent group, respectively (*p* = 0.684). Severe calcified lesions involving more than 180 degrees of arterial circumference comprised 38.5% in the PA group and 35.4% in the PTA ± stent group, respectively (*p* = 0.063). Concomitant aortoiliac and below-the-knee lesions were present at 32.1% and 45.4%, respectively. There was no difference in concomitant AI (*p* = 0.101) and below-the-knee (*p* = 0.192) lesions in the two groups.

### 3.3. Procedure Details

The database of the registry included the detailed procedures for the used balloon and stent diameter, the use of an embolic protection device (EPD) or closure device, and procedure outcomes. The analyzed procedure details are shown in Table 3. In the PA group, rotational atherectomy was used in 39 patients (43.3%) and directional atherectomy was used in 51 patients (56.7%). Re-entry devices were used in only 5 patients (1.2%) in the PTA and/or stenting group. Balloon catheters with diameters of 6–7 mm were used at 54.2% in the PA group and 32.1% in the PTA ± stent group, respectively (*p* = 0.041). Balloon inflation pressure of more than 10 atmospheres comprised 92.8% in the PA group and 54.8% in the PTA ± stent group, respectively (*p* = 0.007). Residual stenosis of more than 50% was present at 15.8% in the PA group and 1.0% in the PTA ± stent group, respectively (*p* = 0.017). The DCB was used in 180 (42.5%) patients, with more common use in the PA group (58.9% vs. 38.0%, *p* < 0.001). The bailout stenting rate was 3.3% in the PA group and 43.4% in the PTA ± stent group, respectively, with statistical significance (*p* < 0.001). The EPD was used in only the PA group. At the completion of the procedure, the closure device for the access site was used at 80.0% in the PA group and 38.6% in the PTA ± stent group, respectively (*p* < 0.001).

Devices used were: atherectomy—Jetstream™ (Boston Scientific, Marlborough, MA, USA), SilverHawk^®^, TurboHawk™, and HawkOne™ (all Medtronic, Minneapolis, MN, USA); stents—Zilver^®^ PTX (Cook Medical, Bloomington, IN, USA), Supera™ (Abbott Vascular, Santa Clara, CA, USA), Eluvia™ (Boston Scientific, Marlborough, MA, USA), and EverFlex™ (Medtronic, Minneapolis, MN, USA); drug-coated balloons—IN.PACT™, Admiral™ (Medtronic, Minneapolis, MN, USA), Ranger™ (Boston Scientific, Marlborough, MA, USA), and Lutonix^®^ (BD, Franklin Lakes, NJ, USA).

### 3.4. Primary Patency Rate of the Propensity-Matched Cohorts

**Figure 1:**
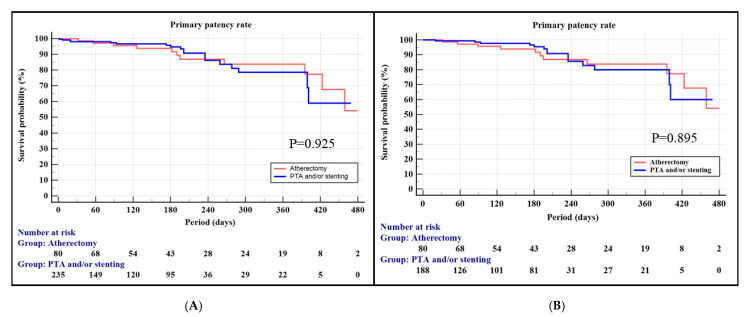
Kaplan–Meier curve for the primary patency rate: (**A**) before matching and (**B**) after matching.

Propensity score matching was performed to minimize the effect of baseline demographics. The demographics after propensity score matching are shown in Table 4. The demographics were similar in the two groups after matching. Primary patency rates (PPRs) before and after the propensity scores were similar in the two groups (Figure 1). The overall one-year PPR was 83.8% in the PA group and 78.6% in the PTA ± stent group (*p* = 0.925). The one-year PPR was not statistically significant in the two groups after propensity score matching (83.8% vs. 80.0%), respectively (*p* = 0.895). Target lesion revascularization (TLR) data were available for 287 patients (atherectomy n = 80; PTA ± stent n = 207). TLR occurred in 4 (5.0%) vs. 5 (2.4%) patients, respectively. Kaplan–Meier estimates of freedom from TLR showed no significant difference between groups (log-rank *p* = 0.266). In the PA group, rotational devices were used in 65 (72.7%) and directional devices in 25 (27.3%) cases. Primary patency did not differ significantly between the two device types (log-rank *p* = 0.503).

### 3.5. Functional and Safety Outcomes

Table 5 demonstrates the functional outcomes, such as ABI and WIQ scores, and safety outcomes after the procedures. Compared with the PTA ± stent group, the preoperative ABI was higher in the PA group (0.47 ± 0.26 vs. 0.36 ± 0.31; *p* = 0.003). However, postoperative ABIs increased with statistical significance in the two groups (*p* < 0.001). The preoperative WIQ scores of the two groups were similar. In addition, postoperative WIQ scores were significantly improved in two groups (*p* < 0.001). Amputation rates, including minor and major types, were 6.8% during the follow-up. The PA group demonstrated a higher amputation rate compared with the PTA ± stent group (12.2% vs. 5.4%: *p* = 0.032). When classified by level, the majority of amputations were minor. Major amputations occurred only in the PTA ± stent group (2 patients, 0.6%), and none occurred in the atherectomy group (0%), with no statistically significant difference between groups (*p* >0.999). The mortality rates of the two groups were similar (2.2% in the PA group vs. 3.0% in the PTA ± stent group; *p* = 695). The detailed causes of death in the two groups are shown in Table 5. There was no procedure-related death.

### 3.6. Hazard Ratio

**Figure 2:**
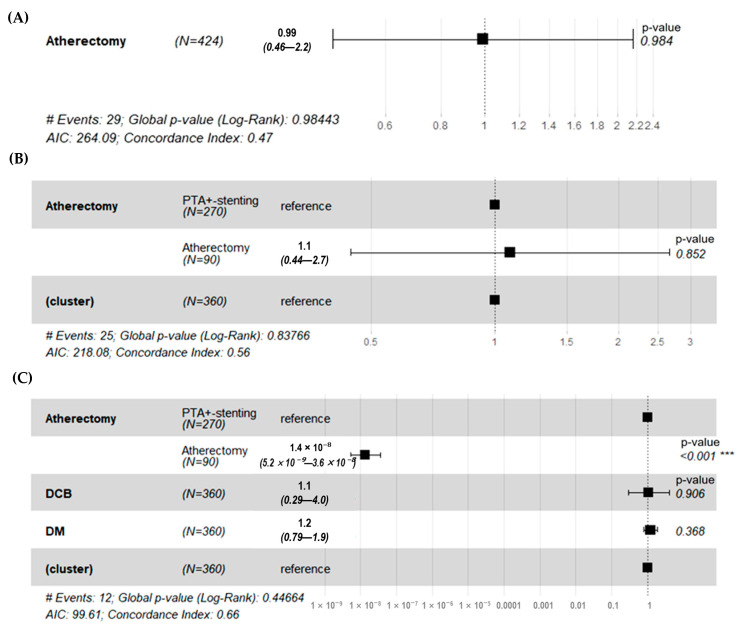
The risk of occlusion after procedure during the follow-up. (**A**) Before matching, the atherectomy group did not see a decrease in the risk of occlusion from the reference of the PTA ± stent group (HR 0.99, *p* = 0.984). (**B**) After 1:3 propensity matching, univariate analysis demonstrated that the atherectomy group did not see a decrease in the risk of occlusion from the reference of the PTA ± stent group (HR 1.1, *p* = 0.852) (**C**) After 1:3 propensity matching, multivariate analysis demonstrated that the atherectomy group saw a significant decrease in the risk of occlusion from the reference of the PTA ± stent group (HR < 0.01, *** *p* < 0.001). However, use of a DCB or diabetes did not affect the risk of occlusion. PTA ± stent = balloon angioplasty and/or stenting; HR = hazard ratio; DCB = drug-coated balloon.

Further analysis was performed to evaluate the effect of DCB use, diabetes status, and atherectomy using a Cox proportional hazard regression model (Figure 2). Before the propensity matching, the atherectomy group did not see a decrease in the risk of occlusion during the follow-up from the reference of the PTA ± stent group (HR 0.99, *p* = 0.984) (Figure 2A). After 1:3 propensity matching, univariate analysis also demonstrated that the atherectomy group did not see a decrease in the risk of occlusion (HR 1.1, *p* = 0.852) (Figure 2B). However, multivariate analysis demonstrated that the atherectomy group significantly decreased the risk of occlusion (HR < 0.01, *p* < 0.001). Furthermore, use of a DCB or diabetes did not affect the risk of occlusion (Figure 2C).

## 4. Discussion

This prospective registry study demonstrates the comparison outcomes between PA and PTA ± stent for femoropopliteal lesions from the nationwide DAMOEUM database. This registry included the detailed anatomic characteristics and procedure details of each patient. Atherectomy could not increase the PPR until 480 days. However, atherectomy reduced the need for bailout stenting and the incidence of occlusion during the follow-up in femoropopliteal arterial diseases. Contemporary multicenter registry data (XLPAD) demonstrate substantial heterogeneity in real-world outcomes and reintervention rates after femoropopliteal endovascular therapies, highlighting the influence of lesion complexity and treatment selection on durability [12].

The burden of intra-arterial plaque is one of the significant factors influencing the outcomes after femoropopliteal intervention. Patel SD et al. reported a strong association between the percentage of calcified plaque and both binary restenosis and reintervention rate, as well as a negative correlation between the total volume of calcified plaque and amputation-free survival [13]. Therefore, theoretically, the patency rate can be improved by reducing the intra-arterial plaque through the PA. Taneva GT et al. reported promising mid-term outcomes after the use of rotational atherectomy for the treatment of severe femoropopliteal diseases and showed relatively low need for bailout stenting and good mid-term primary patency rates [14]. The PPRs were 97% at 12 months and 83% at 24 months, with secondary patency rates of 99% at 12 months and 91% at 24 months of follow-up. In addition, Noory E et al. analyzed the 2-year outcomes after use of directional atherectomy and front-cutting atherectomy for the treatment of atherosclerotic lesions of the femoropopliteal arteries [10]. The two-year target lesion revascularization (TLR)-free survival for de novo lesions was 23.1%. However, this study showed a lower one-year patency rate and no difference in PPR compared to balloon angioplasty alone. Several meta-analysis studies demonstrated similar results with this study. Wu Z et al. performed a systematic review and meta-analysis comparing the outcomes between balloon angioplasty alone and atherectomy combined with balloon angioplasty for patients with de novo femoropopliteal steno-occlusive lesions [15]. Atherectomy combined with BA may not improve primary patency, TLR, mortality rate, or ABI [15,16]. Lin F et al. compared the outcomes after a DCB only with those after atherectomy plus a DCB for the treatment of femoropopliteal artery lesions using six studies including two randomized controlled trials and four retrospective cohort studies [17]. There was no significant difference between the two groups in terms of the PPR at 12 months. One of the biggest reasons for showing the differences between studies is postulated with the differences in the lesion types in the studies. This registry study included all types of lesion, including de novo lesion, restenotic lesion, and repeated intervention. Restenotic and repeated interventional lesions showed significant unfavorable outcomes [10].

Severe calcification involving more than 180° of the vessel circumferences was observed in approximately 50% of patients in the PA group. The atherectomy procedure was effective in increasing lumen dimensions in moderately or severely calcified femoropopliteal lesions by removing superficial calcium [18]. Intravascular ultrasound (IVUS) plays a valuable role in treating calcified femoropopliteal lesions, providing detailed imaging to assess the extent and pattern of calcification, which can optimize endovascular therapy [19,20]. Even when an IVUS catheter cannot cross the lesion, it offers critical information just proximal to the target lesion, aiding in the selection of appropriate guidewire and burr sizes [21]. Further studies are needed to evaluate the efficacy of atherectomy with the concomitant use of IVUS.

Balloon diameter is an important factor in improving outcomes after percutaneous intervention, as achieving a larger luminal diameter with an appropriately sized balloon is a key predictor of patency [22]. In the PA group, balloons with diameters greater than 6 mm were used in 54.2% of cases, compared with 32% in the PTA ± stent group. This higher usage of larger balloons in the PA group is likely due to the reduced rate of flow-limiting dissections after atherectomy. Atherectomy has been shown to reduce significant dissections in treating de novo femoropopliteal stenosis–occlusive disease following balloon angioplasty [23]. Balloon inflation pressure also plays a role in optimizing outcomes. Atherectomy prepares vessels by debulking plaque burden, often allowing for revascularization with lower PTA pressures, which can decrease the risk of intimal hyperplasia and dissection [22]. In this study, balloon pressure tended to be higher in the PA group. Although this may partly reflect differences in lesion characteristics, the luminal gain achieved with atherectomy likely facilitates balloon expansion to the desired pressure. Interestingly, residual stenosis was somewhat higher in the atherectomy group. This likely reflects case selection, as atherectomy was preferentially used in complex and calcified lesions, where operators often accepted higher residual narrowing to avoid aggressive balloon dilatation and thereby reduce the risk of dissection or perforation.

This study revealed that the incidence of bailout stenting was significantly lower in the PA group than in the PTA ± stent group (3.3% vs. 43.4%). It has been demonstrated in previous studies that the mechanism of action involves calcified plaque modification and increased luminal gain, resulting in fewer dissections [23,24].

In this study, the incidence of amputation was 12.2% in the PA group. The amputation data included both minor and major amputations, and the higher incidence may partly reflect the inclusion of planned minor amputations. Another contributing factor may be the higher percentage of severe calcification in the PA group, as distal embolization is more likely to occur when treating heavily calcified lesions. Severe calcification was observed more frequently in the PA group compared with the PTA ± stent group (50% vs. 35.4%). Despite the use of EPDs during atherectomy, distal embolization remains a potential limitation of this approach [25,26]. Because embolization can lead to serious clinical consequences, using EPDs in high-risk patients is recommended, as these devices are known to reduce the need for repeat interventions and mitigate clinical sequelae [27].

One important finding of this study is that no cases of complete occlusion were observed in the PA group during the follow-up. Although percutaneous intervention for femoropopliteal lesions is effective, it has drawbacks, such as the need for reintervention due to elastic recoil or late lumen loss. Restenosis is a primary factor affecting the long-term patency of femoropopliteal lesions. Several studies have identified factors closely associated with restenosis, including severe calcification, long-segment occlusion, and in-stent restenosis, which are the characteristics of femoropopliteal lesions [28]. Reinterventions for restenosis can be more challenging than interventions for de novo lesions, as treating complete occlusions increases the complexity of the procedure and the risk of complications [29]. When considering reintervention, it is crucial to successfully pass the guidewire through the lesion. If the lesion is stenotic rather than occlusive, reintervention can be relatively straightforward. One significant advantage for the use of an atherectomy device is its ability to facilitate this process.

Our study has several limitations. First, data loss occurred among some patients due to the use of consecutively collected registry data. Additionally, the analysis included both minor and major amputations without distinguishing between the levels. Since the data were collected from multiple centers, surgeries were performed according to each physician’s individual methods rather than following a standardized protocol. Despite this, the inclusion of data from multiple centers enhances the generalizability of our findings. Moreover, the detailed patient characteristics and procedure-specific information in the registry allowed for in-depth analysis.

This study aimed to evaluate the role of endovascular atherectomy by comparing primary patency to other endovascular treatments (EVTs) for femoropopliteal lesions. Currently, there is no established decision algorithm for selecting the optimal treatment strategy due to concerns about stent fractures and restenosis in the high-biomechanical-stress region of the femoropopliteal region [30]. Our study aligns with previous research indicating that atherectomy does not significantly alter the long-term outcomes of femoropopliteal arterial disease treatment compared to PTA alone [15,31]. In our study, atherectomy did not provide significant additional clinical benefit in terms of patency rates. The use of IVUS is recommended to reduce restenosis rates [32]. However, it is not currently reimbursed in Korea, leading to suboptimal treatment and comparable outcomes to other EVT options.

Despite these findings, our study indirectly provided evidence regarding factors influencing patency improvement during atherectomy by matching patients based on propensity scores to minimize the impact of demographic characteristics. When comparing the matched cohort, we observed no significant differences in the primary outcomes between the PA and PTA ± stent groups. However, when considering the effect of a DCB, atherectomy showed a significantly lower risk of occlusion. Nonetheless, further data are required to support the use of DCBs after controlling for atherectomy.

Another noteworthy observation in our study was the significantly lower stenting rate (3.3% vs. 45.6%) in the PA group. This suggests that many vascular surgeons prefer atherectomy to avoid stenting in the treatment of femoropopliteal occlusive disease. This preference may potentially benefit patients by making rescue procedures less challenging in cases of restenosis or reocclusion. Additionally, patients who received atherectomy showed higher pre-procedural ABI values compared with those who received other types of EVT, indicating the preference of vascular surgeons for atherectomy in certain cases.

However, this study has limitations inherent to its observational nature and the involvement of multiple individuals in data entry, which could introduce coding errors, missing data, and a lack of anatomical details. TLR, although a key endpoint, could not be fully analyzed because data were missing in approximately one-third of patients; we therefore reported only the available cases, and results should be interpreted with caution. In addition, information on whether amputations were planned or unplanned was not available, lesion length was not captured in the registry, and subgroup analyses such as directional versus rotational atherectomy were limited by small sample sizes. Nevertheless, the strength of this study lies in its use of a multicenter database collected nationwide, providing novel data specific to the Korean population and facilitating comparisons with other ethnic groups.

## 5. Conclusions

In this nationwide multicenter registry, the use of atherectomy was not associated with improvement in target lesion patency compared to PTA with or without stenting. However, atherectomy was associated with a significantly lower bailout stenting rate and reduction in target lesion occlusion during follow-up. Major amputations were rare and overall adverse event rates, including target lesion revascularization, were low in both groups. Taken together, these findings suggest that atherectomy may be selected as a preferred modality in patients where repeated intervention is anticipated or when minimizing stent implantation is desirable. Nevertheless, given the observational design and missing data for some endpoints, further prospective studies are needed to validate these findings.

## Figures and Tables

**Table 1:** Baseline demographics.

Characteristics	All (N = 424)	Atherectomy (n = 90)	PTA ± Stenting (n = 334)	*p* Value
Age, years	72 (66–78)	69 (64–75)	72 (66–79)	0.016
Male gender	345 (81.4)	77 (85.6)	268 (80.2)	0.137
Body mass index, kg/m^2^	23 (21–25)	23 (22–25)	23 (21–25)	0.306
Hypertension	329 (77.6)	71 (78.9)	258 (77.2)	0.999
Diabetes	266 (62.7)	56 (62.2)	210 (64.8)	0.709
Chronic kidney disease	136 (32.1)	26 (29.5)	110 (34.0)	0.523
Hyperlipidemia	200 (48.3)	45 (50.0)	155 (47.8)	0.722
Coronary artery disease	139 (34.2)	33 (36.7)	106 (33.5)	0.615
Pulmonary disease	118 (29.3)	39 (43.3)	79 (25.2)	0.001
Smoking				0.651
Ex-smoker	43 (10.4)	3 (3.4)	40 (12.3)	
Current smoker	124 (29.2)	28 (31.5)	96 (29.4)	
Medication				
Antiplatelet	269 (63.4)	64 (71.1	205 (61.4)	0.001
Anticoagulant	38 (9.0)	9 (10.0)	29 (8.7)	0.001
Rutherford category				<0.001
Claudication	167 (39.4)	58 (64.4)	109 (31.7)	
Rest pain	142 (23.5)	27 (30.0)	125 (36.3)	
Minor tissue loss	62 (14.6)	4 (4.4)	58 (16.9)	
Major tissue loss	53 (12.5)	1 (1.1)	52 (14.5)	
WIQ score	51 (33–66)	55 (37–69)	50 (31–64)	0.106
ASA classification	3 (2–3)	3 (2–3)	3 (2–3)	0.125

PTA, percutaneous transluminal angioplasty; WIQ, Walking Impairment Questionnaire; ASA, American Society of Anesthesiologists. Data are presented as medians (interquartile range) or numbers (%).

**Table 2:** Anatomic characteristics.

Characteristics	All (N = 424)	Atherectomy (n = 90)	PTA ± Stenting (n = 334)	*p* Value
Lesion type				0.725
De novo	333 (78.5)	69 (76.7)	264 (79.0)	
In-stent restenosis	41 (9.7)	7 (7.8)	34 (10.2)	
Re-intervention	39 (9.2)	7 (7.8)	32 (9.6)	
Lesion site				0.745
Proximal SFA	190 (44.8)	50 (55.6)	140 (41.9)	
Mid-SFA	217 (51.2)	45 (50.0)	172 (51.5)	
Distal SFA	204 (48.1)	48 (53.3)	156 (46.7)	
P1	117 (27.6)	29 (32.2)	88 (26.3)	
P2	60 (14.2)	13 (14.4)	47 (14.1)	
P3	35 (8.3)	6 (6.7)	29 (8.7)	
TASC classification				0.684
TASC A	77 (18.2)	17 (18.9)	60 (18.0)	
TASC B	198 (46.7)	42 (46.7)	156 (46.7)	
TASC C	65 (15.3)	16 (17.8)	49 (14.7)	
TASC D	64 (15.1)	14 (15.6)	50 (15.0)	
Calcium grade				0.063
No calcification	91 (21.5)	16 (17.8)	75 (22.5)	
Circumference 1°~89°	82 (19.3)	17 (18.9)	65 (19.5)	
Circumference 90°~179°	42 (9.9)	8 (8.9)	34 (10.2)	
Circumference 180°~269°	49 (11.6)	11 (12.2)	38 (11.4)	
Circumference 270°~360°	114 (26.9)	34 (37.8)	80 (24.0)	
Concomitant inflow lesions				0.101
None	288 (67.9)	67 (74.4)	221 (66.2)	
Acute	59 (13.9)	14 (15.6)	45 (13.5)	
Chronic	77 (18.2)	9 (10.0)	68 (20.4)	
Concomitant outflow lesions				0.192
None	230 (54.2)	53 (58.9)	177 (53.0)	
Acute	95 (22.4)	19 (21.1)	76 (22.8)	
Chronic	99 (23.3)	18 (20.0)	81 (24.3)	

PTA, percutaneous transluminal angioplasty; SFA, superficial femoral artery; *p*, popliteal artery; P1 corresponds to the proximal segment, from the channel of the adductor muscles to the upper border of the patella; P2 is the middle segment, from the upper border of the patella to the joint line; P3 corresponds to the distal segment, from the joint line to the emergence of the anterior tibial artery; TASC, TransAtlantic InterSociety Consensus Data, which are presented as numbers (%).

**Table 3:** Procedure details.

Characteristics	All (N = 424)	Atherectomy (n = 90)	PTA ± Stenting (n = 334)	*p* Value
Use of re-entry device	5 (1.2)	0	5 (1.5)	0.589
Balloon diameter (n = 242)				0.041
4–5.5 mm	159 (65.7)	11 (45.8)	148 (67.9)	
6–7 mm	83 (34.3)	13 (54.2)	70 (32.1)	
Balloon inflation pressure (n = 118)				0.007
<10 atm	48 (40.7))	1 (7.1)	47 (45.2)	
10–15 atm	66 (55.9)	12 (85.7)	54 (51.9)	
>15–20 atm	4 (3.4)	1 (7.1)	3 (2.9)	
Residual stenosis (n = 211)				0.017
<30%	192 (91.0)	16 (84.2)	176 (91.7)	
30~50%	14 (6.6)	0	14 (7.3)	
>50%	5 (2.4)	3 (15.8)	2 (1.0)	
Use of the drug-coated balloon	180 (42.5)	53 (58.9)	127 (38.0)	<0.001
Stent placement	148 (34.9)	3 (3.3)	145 (43.4)	<0.001
Stent diameter				0.646
4 mm	2 (1.4)	0	2 (1.4)	
5 mm	33 (22.3)	0	33 (22.8)	
6 mm	99 (66.9)	3 (100)	96 (66.2)	
7 mm	14 (9.5)	0	14 (9.7)	
Use of embolic protection device	83 (19.6)	83 (92.2)	0	<0.001
SpiderFX	77 (92.8)	77 (92.8)	0	
Emboshield	6 (7.2)	6 (7.2)	0	
Use of closure device	201 (47.4)	72 (80.0)	129 (38.6)	<0.001

PTA, percutaneous transluminal angioplasty; atm, atmosphere. Data are presented as numbers (%).

**Table 4:** Patient characteristics after 1:3 propensity-matched cohorts.

Characteristics	Atherectomy (n = 90)	PTA ± Stenting (n = 270)	*p* Value
Age, years	69 (64–75)	71 (66–79)	0.066
Male gender	77 (85.6)	215 (79.6)	0.137
Body mass index, kg/m^2^	23.2 (21.6–25.0)	22.7 (20.6–25.0)	0.294
Hypertension			0.159
Controlled with 1 drug	26 (28.9)	102 (37.8)	
Controlled with 2 drugs	30 (33.3)	80 (29.6)	
Requires more than two drugs or is uncontrolled	15 (16.7)	29 (10.7)	
Diabetes mellitus			0.052
Controlled by oral drug	34 (14.4)	94 (34.8)	
Controlled by insulin	6 (6.7)	19 (7.0)	
Type I diabetes or uncontrolled	37 (41.1)	65 (24.1)	
Smoking			0.936
Ex-smoker	3 (3.4)	29 (10.7)	
Current smoker (<1 pack/day)	14 (15.7)	44 (16.3)	
Current smoker (≥1 pack/day)	14 (15.7)	35 (13.0)	
Indication			0.077
Intermittent claudication	49 (54.4)	118 (43.7)	
Rest pain	34 (37.8)	102 (37.8)	
Minor tissue loss	5 (4.7)	41 (15.2)	
Major tissue loss	2 (2.2)	9 (3.3)	

PTA, percutaneous transluminal angioplasty. Data are presented as medians (interquartile range) or numbers (%).

**Table 5:** Functional and safety outcomes.

Characteristics	All (N = 424)	Atherectomy (n = 90)	PTA ± Stenting (n = 334)	*p* Value
Ankle–brachial index				
Pre-procedure	0.41 ± 0.29	0.47 ± 0.26	0.36 ± 0.31	0.003
Post-procedure	0.90 ± 0.24	0.94 ± 0.24	0.91 ± 0.24	0.340
*p* value	<0.001	<0.001	<0.001	
Walking Impairment Questionnaire score				
Pre-procedure	51.9 ± 25.0	52.3 ± 22.4	46.1 ± 25.8	0.106
Post-procedure	86.3 ± 16.4	87.5 ± 12.9	85.8 ± 17.8	0.523
*p* value	<0.001	<0.001	<0.001	
Amputation rate	29 (6.8)	11 (12.2)	18 (5.4)	0.032
Mortality rate	12 (2.8)	2 (2.2)	10 (3.0)	0.695
Cause of death	3 Myocardial infarction 2 Pneumonia 1 ARDS 1 Cancer progression 1 COVID 1 Hydropneumothorax 1 Ischemic colitis 1 Sepsis 1 Subdural hemorrhage	1 COVID 1 Pneumonia	3 Myocardial infarction 1 ARDS 1 Cancer progression 1 Hydropneumothorax 1 Ischemic colitis 1 Pneumonia 1 Sepsis 1 Subdural hemorrhage	

PTA, percutaneous transluminal angioplasty; ARDS, acute respiratory distress syndrome; COVID, coronavirus disease-19. Data are presented as numbers (%) and means ± standard deviation.

## Data Availability

The de-identified dataset underlying the findings of this study, together with a data dictionary and analysis code, will be available on the article webpage upon publication.

## Abbreviations

CLTI	Chronic limb-threatening ischemia
PA	Percutaneous atherectomy
DCB	Drug-coated balloon
US	United States
KSVS	Korean Society for Vascular Surgery
WIQ	Walking Impairment Questionnaire
TASC	TransAtlantic InterSociety Consensus
SFA	Superficial femoral artery
CT	Computed tomogram angiography
MRA	Magnetic resonance image angiography
ABI	Ankle–brachial index
HR	Hazard ratio
CI	Confidence interval
EPD	Embolic protection device
PPR	Primary patency rate
TLR	Target lesion revascularization
IVUS	Intravascular ultrasound
EVT	Endovascular treatment
